# Intersectoral and multisectoral approaches to health policy: an umbrella review protocol

**DOI:** 10.1186/s12961-022-00826-1

**Published:** 2022-02-15

**Authors:** Michelle Amri, Ali Chatur, Patricia O’Campo

**Affiliations:** 1 Dalla Lana School of Public Health, University of Toronto, 155 College St., Toronto, ON M5T 1P8 Canada; 2 Takemi Program in International Health, Harvard University, Boston, USA; 3 Health Studies, University College, University of Toronto, Toronto, Canada; 4 Li Ka Shing Knowledge Institute, St. Michael’s Hospital, Toronto, Canada

**Keywords:** Health in All Policies, HiAP, Healthy cities, One Health, Multisectoral, Intersectoral, Health, Governance, Healthy public policy, Implementation

## Abstract

**Background:**

It is widely recognized that one’s health is influenced by a multitude of nonmedical factors, known as the social determinants of health (SDH). The SDH are defined as “the conditions in which people are born, grow, live, work and age, and which are shaped by the distribution of money, power and resources at global, national and local levels”. Despite their influence on health, most of the SDH are targeted through government departments and ministries outside of the traditional health sector (e.g. education, housing). As such, the need for intersectoral and multisectoral approaches arises. Intersectoral and multisectoral approaches are thought to be essential to addressing many global health challenges our world faces today and achieving the Sustainable Development Goals. There are various ways of undertaking intersectoral and multisectoral action, but there are three widely recognized approaches (Health in All Policies [HiAP], Healthy Cities, and One Health) that each have a unique focus. However, despite the widespread acceptance of the need for intersectoral and multisectoral approaches, knowledge around how to support, achieve and sustain multisectoral action is limited. The goal of this study is to assemble evidence from systematic approaches to reviewing the literature (e.g. scoping review, systematic review) that collate findings on facilitators/enablers and barriers to implementing various intersectoral and multisectoral approaches to health, to strengthen understanding of how to best implement health policies that work across sectors, whichever they may be.

**Methods:**

An umbrella review (i.e. review of reviews) is to be undertaken to collate findings from the peer-reviewed literature, specifically from Ovid MEDLINE and Scopus databases. This umbrella review protocol was developed following the preferred reporting items for systematic review and meta-analysis protocols (PRISMA-P), and study design informed by the PRISMA guidelines for scoping reviews (PRISMA-ScR).

**Discussion:**

Countries that employ multisectoral approaches are better able to identify and address issues around poverty, housing and others, by working collaboratively across sectors, with multisectoral action by governments thought to be *required* to achieve health equity.

## Background

There is wide recognition that one’s health is influenced by a multitude of nonmedical factors, known as the social determinants of health (SDH). The SDH are defined as “the conditions in which people are born, grow, live, work and age, and which are shaped by the distribution of money, power and resources at global, national and local levels” [1]. These SDH (termed differently depending on the author or institution but remain largely the same) include income and social protection, education, unemployment and job security, working life conditions and others [2, 3]. Despite their influence on health, most of the SDH are targeted through government departments and ministries outside of the traditional health sector. For example, education and housing are largely influenced outside of the scope of health ministries. As such, the need for intersectoral and multisectoral approaches arises.

### Background on intersectoral and multisectoral approaches

Intersectoral and multisectoral approaches are defined as collaborative approaches, which can span across various ministries, government agencies, nongovernmental organizations, relevant stakeholders and other groups, with a common goal in addressing a particular issue [4]. Differing from other non-intersectional methods, intersectoral and multisectoral approaches aim to address the “social and economic factors that influence the health of a population at the local, national, and global levels” [5]. Given the ability of intersectoral and multisectoral approaches to address social and economic factors, and the aforementioned role of the SDH which operate outside of the traditional health sector in influencing health, it is understandable that there is wide recognition that intersectoral and multisectoral approaches are needed in health. Key reports, such as the Ottawa Charter for Health Promotion emerging from the first International Conference on Health Promotion in 1986, bring attention to this by discussing the need to build healthy public policy, stating “health promotion […] puts health on the agenda of policy makers in all sectors and at all levels, directing them to be aware of the health consequences of their decisions and to accept their responsibilities for health” [6].

Similarly, these approaches are widely agreed upon by the health community as needing to be included in the future and for future health planning. Dating back nearly two decades ago, the 1997 Canadian conference “Intersectoral action for health: a cornerstone for health for all in the twenty-first century” discussed how intersectoral health was to be a major part of health and policy planning [7]. More recently, a large regional WHO meeting convened health sector directors of policy and planning from the African Region, who pointed to the need to utilize multisectoral approaches to address primary healthcare as a means for achieving universal health coverage [8]. Discussions at this meeting yielded four key considerations around the importance of intersectoral and multisectoral approaches: (i) to work across the humanitarian and development divide, (ii) because many health issues are “spillovers” from other sectors (e.g. transport and associated road injuries), (iii) the need to work with those yielding different power (e.g. ministries of finance who develop the budget) and (iv) the need to work with politicians, given the political nature of health [8]. These ideas reiterate the need for multisectoral approaches in primary healthcare, as multisectoral approaches are linked to integrating primary healthcare in an ethical and sustainable manner and can promote enhanced aid coordination and public health system strengthening in low- and middle-income countries [9]. In fact, intersectoral and multisectoral approaches are thought to be essential to addressing many global health challenges our world faces today and achieving the Sustainable Development Goals [10].

### Formalized approaches to intersectoral and multisectoral action

There are various ways of undertaking intersectoral and multisectoral action, but there are three widely recognized approaches (Health in All Policies [HiAP], Healthy Cities and One Health), each with a unique focus.

HiAP is “is an approach to public policies across sectors that systematically takes into account the health implication of decisions, seeks synergies, and avoids harmful health impacts in order to improve population health and health equity” [11]. In other words, it seeks to promote action on the SDH and consideration for health in other sectors [12]. An approach based on health rights, this encourages policy-makers to look at health as an integral part of how policy is formed [13].

The Healthy Cities project was designed to “support integrated approaches to health promotion at the city level” [14] and began as a project to encourage cities across Europe to take a more wholesome approach when it comes to addressing health issues [15]. In other words, it aims to put health on the agenda at the municipal level. Healthy Cities implement intersectoral health plans, along with collaborating with other cities to support further Healthy City development and establish networks [14].

And lastly, the One Health approach focuses on the interaction between humans and animals and the associated influence on health [16]. Through the creation and design of programs with a multisectoral approach, the goal is to work collaboratively across sectors to address health issues that are relevant to how health is connected to our surrounding environment. The One Health approach has been gaining attention in recent years: first, through increasing attention paid to antimicrobial resistance, particularly following the convening of the United Nations (UN) General Assembly and associated commitment to act, marking only the fourth time in history that the UN met to discuss a health issue [17]; and second, the onset of the COVID-19 pandemic, which has not only had serious implications for health, but demonstrated weak and limited public policy coordination, the need to engage in strategic planning and prioritizing foresight to address pre-existing policy problems (such as income supports, which lie outside of the health sector), and opportunity for multisectoral and cross-discipline discussions and action [18, 19].

### Current knowledge limitations

Despite the widespread acceptance of the need for intersectoral and multisectoral approaches, knowledge around how to support, achieve and sustain multisectoral action is limited [10]. More research is needed to better understand the silos that exist in the creation of health policy, especially when it comes to implementing these approaches. In the case of the human–animal–environment interface, the need to draw on different disciplinary backgrounds to approach the issue as one holistic problem, rather than as separate issues, has been raised [20]. And while there have been studies that seek to collate evidence on multisectoral action with a specific focus (e.g. HiAP), we postulate that successes in working cross-sectorally to achieve health goals with one approach can glean insights and perhaps translate to other approaches which work across sectors (i.e. shared insights across HiAP, Healthy Cities, One Health and other approaches) [21]. At present, we are unaware of any analysis or studies (e.g. umbrella reviews, reviews of reviews) that seek to collate findings from across intersectoral and/or multisectoral approaches.

## Methods

### Aim of the study

The goal of this study is to assemble evidence from systematic approaches to reviewing the literature (e.g. scoping review, systematic review) that collate findings on facilitators/enablers of and barriers to implementing various intersectoral and multisectoral approaches to health, to strengthen understanding of how to best implement health policies that work across sectors, whichever they may be. By undertaking an umbrella review (i.e. review of reviews), the aim is to provide a rigorous evidence base for policy-makers to inform intersectoral and multisectoral approaches and, in doing so, to advance the literature to clarify priorities for further investigation. Given the various approaches to working intersectorally and multisectorally (i.e. HiAP, Healthy Cities, One Health and others that are not characterized by these terms), this study approaches the domain with a broad scope. We hope we can apply this broad scope to cast a wide net, but intentionally searched across these three approaches to improve the accuracy of our search.

### Study design

Seeking to collate findings from the peer-reviewed literature, an umbrella review (i.e. review of reviews) is to be undertaken. An umbrella review compiles evidence from numerous reviews to develop an accessible document [22]. More specifically, an umbrella review summarizes across research syntheses; it does not re-summarize existing reviews [23]. In the case of this study, this umbrella review aggregates evidence on facilitators/enablers of and barriers to implementing intersectoral and multisectoral action across varying approaches to best inform future action.

This umbrella review protocol was developed following the preferred reporting items for systematic review and meta-analysis protocols (PRISMA-P) [24], and the study design was informed by the PRISMA guidelines for scoping reviews (PRISMA-ScR) [25].

### Search strategy

Given the focus on intersectoral and multisectoral approaches, both Ovid MEDLINE (a medical science database) and Scopus (an interdisciplinary database) will be used to conduct the search. This aligns with the aim of umbrella reviews set out by Smith et al. [26], which entails being “comprehensive, thorough, and objective”. The search was conducted on 19 September 2020 and does not specify a starting time frame (i.e. from database inception), using the following search string: (“one health” OR “health in all polic*” OR “HiAP” OR “healthy cit*” OR “intersectoral” OR “multisectoral”) AND “health*” AND “review” AND “implement*”.

### Inclusion criteria

The inclusion criteria eligibility is as follows: must be a review (e.g. scoping, systematic) that systematically (i.e. empirically) collates findings from sources around facilitators/enablers of and/or barriers to implementation of intersectoral/multisectoral policy/governance approaches to health. Manuscripts that have both a systematic approach to literature and quantitative and/or qualitative data analysis component will also be included.

### Exclusion criteria

Documents are excluded if they are (i) not focused on policy/governance (e.g. interprofessional collaboration in clinical care, patient records for telemedicine) or (ii) not available in English (due to resource constraints).

### Data management

Extracted references will be imported into EndNote X9 software. Utilizing Covidence software, duplicates will be removed, and the first screen (titles and abstracts) of references will be undertaken by two independent reviewers. Conflicts during the first screen will be discussed by the two independent reviewers to collaboratively resolve. Following the first screen, articles marked for full-text review will be read by both reviewers to ensure alignment with the inclusion criteria (and not falling into the exclusion criteria). Any discrepancies will be reviewed by the full authorship team, who will accordingly determine the final articles included in the study.

### Data charting and analysis

Data extracted from the full texts will be charted to detail more bibliographic information (authors [year]; article title; years covered in the review; number of studies included in the review; cities and/or countries; aim or objective; and specific to One Health, HiAP, Healthy Cities, or general?) and the facilitators/enablers and barriers identified by study (article title, facilitators/enablers, barriers). The facilitators/enablers and barriers will also be narratively synthesized by drawing on qualitative methods, specifically thematic analysis. Included articles will be reread in full and coded both deductively, using a priori codes of “facilitators/enablers” and “barriers”, and inductively based on themes of specific facilitators/enablers and barriers. NVivo software will be used to conduct coding and facilitate analysis, which will be led by one reviewer and reviewed by a second. Potential discrepancies in the charting process and analysis will be discussed and agreed upon by the full authorship team. Any amendments to the study protocol will be submitted to the journal and recounted in the final study manuscript.

### Alignment with umbrella review method

These inclusion criteria and broader approach were agreed upon through deliberation with the authorship team, which possesses both experience in conducting systematic reviews of the literature and knowledge of the topic, both of which have been called for in umbrella reviews by Smith et al. [26]. While there is the AMSTAR tool [27] for assessing the quality of systematic reviews for inclusion in an umbrella review, our focus on collating broader systematic approaches to literature reviews (i.e. not just systematic reviews but also scoping reviews) meant that this is not directly applicable. However, key domains of the tool were considered and will be used, similar to the work of Smith et al. [26]. For example, in designing our study, we ensured that two databases were searched and that studies to be collated would be compared based on the outcomes of interest.

In addition, we will seek to present the major conclusions of the umbrella review in alignment with the research question posed [26]. Therefore, our discussion will group findings under facilitators/enablers and barriers. Similarly, for ease of use by policy-makers and to supplement text, we anticipate summarizing these in a table to highlight overarching facilitators/enablers and barriers.

### Limitations

While this study aims to employ a systematic and rigorous search process, only articles available in English are to be included due to resource constraints. However, the inclusion of only English-language articles poses a limitation because it has the potential to exclude research and analysis not written in English.

## Conclusion

Because health is political, policy and governance must be focused on and better addressed [28]. Countries that employ multisectoral approaches are better able to identify and address issues around poverty, housing and other aspects, by working collaboratively across sectors [29]. In fact, it is believed that multisectoral action by governments is *required* to achieve health equity [30]; the Ottawa Charter further supports this view, stating that “it is coordinated action that leads to health, income and social policies that foster greater equity” [6].

While the benefits of intersectoral and multisectoral strategies are apparent, the information on how to effectively *implement* the practices is not widely available, beyond individual studies on specific approaches (e.g. HiAP). And because evidence can ignite action, it is crucial to accord deliberate attention to what constitutes evidence [31]. As such, this umbrella review aims to collate these findings around how to best implement intersectoral and multisectoral approaches, to assist policy-makers, government officials and other relevant stakeholders in implementing intersectoral and multisectoral approaches. The goal is to promote multisectoral collaboration, which “remains untapped in many low- and middle-income countries” [32].

## Data Availability

Data sharing is not applicable to this article as no datasets were generated or analysed during the current study.

## Abbreviations

HiAP: Health in All Policies
SDH: Social determinants of health
UN: United Nations
